# Substrates for Expansion of Corneal Endothelial Cells towards Bioengineering of Human Corneal Endothelium

**DOI:** 10.3390/jfb6030917

**Published:** 2015-09-11

**Authors:** Jesintha Navaratnam, Tor P. Utheim, Vinagolu K. Rajasekhar, Aboulghassem Shahdadfar

**Affiliations:** 1 Department of Ophthalmology, Oslo University Hospital, Postbox 4950 Nydalen, Oslo 0424, Norway; E-Mail: aboulghassem.shahdadfar@medisin.uio.no; 2 Department of Medical Biochemistry, Oslo University Hospital, Postbox 4950 Nydalen, Oslo 0424, Norway; E-Mail: utheim2@gmail.com; 3 Department of Oral Biology, Faculty of Dentistry, University of Oslo, Postbox 1052, Blindern, Oslo 0316, Norway; 4 Memorial Sloan Kettering Cancer Center, Rockefeller Research Building, Room 1163, 430 East 67th Street/1275 York Avenue, New York, NY 10065, USA; E-Mail: Vinagolr@mskcc.org

**Keywords:** cell sources, corneal endothelium, human corneal endothelial cells, substrates, tissue engineering

## Abstract

Corneal endothelium is a single layer of specialized cells that lines the posterior surface of cornea and maintains corneal hydration and corneal transparency essential for vision. Currently, transplantation is the only therapeutic option for diseases affecting the corneal endothelium. Transplantation of corneal endothelium, called endothelial keratoplasty, is widely used for corneal endothelial diseases. However, corneal transplantation is limited by global donor shortage. Therefore, there is a need to overcome the deficiency of sufficient donor corneal tissue. New approaches are being explored to engineer corneal tissues such that sufficient amount of corneal endothelium becomes available to offset the present shortage of functional cornea. Although human corneal endothelial cells have limited proliferative capacity *in vivo*, several laboratories have been successful in *in vitro* expansion of human corneal endothelial cells. Here we provide a comprehensive analysis of different substrates employed for *in vitro* cultivation of human corneal endothelial cells. Advances and emerging challenges with *ex vivo* cultured corneal endothelial layer for the ultimate goal of therapeutic replacement of dysfunctional corneal endothelium in humans with functional corneal endothelium are also presented.

## 1. Introduction

**Figure 1:**
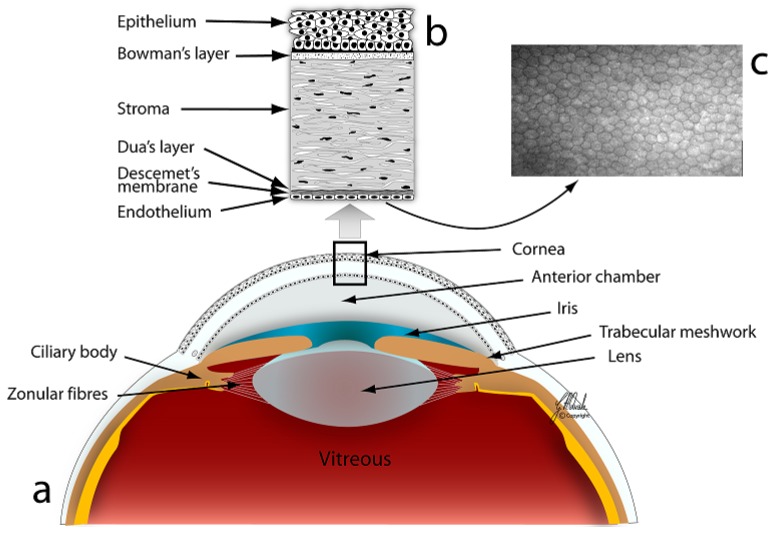
Anatomy of the cornea. (**a**) Section of the anterior part of the eye; (**b**) Section of the cornea illustrating six layers; (**c**) *In vivo* confocal microscopy image of the corneal endothelium. Courtesy of Geir A. Qvale.

The cornea is the transparent anterior part of the eye that transmits and focuses light onto the retina. From anterior to posterior (Figure 1), the cornea is composed of the corneal epithelium (50 μm thick), the Bowman’s membrane (12 μm), the stroma (480–500 μm), the Descemet’s membrane (8–10 μm), and the endothelium (5 μm) [1]. Recently, a new layer of the cornea, Dua’s layer, was also described [2].

**Figure 2:**
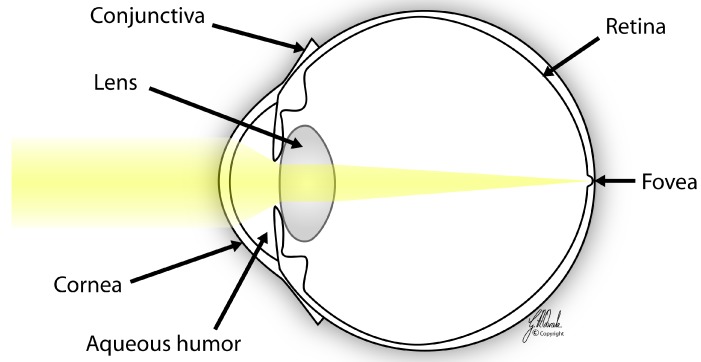
The refraction of light. The cornea provides more than two-thirds of the total refractive power of the eye. Courtesy of Geir A. Qvale.

The human cornea has a thickness of 0.5–0.6 mm centrally and 0.6–0.8 mm peripherally [3]. The horizontal diameter of an average adult human cornea is 11.7 mm and the vertical diameter is approximately 1 mm less than the horizontal diameter. The cornea is one of the few avascular tissues in the body. The cornea is also one of the most heavily innervated and sensitive tissues in the body, with a density of nerve endings about 300–400 times greater than the skin [1,4], thus diseases of the cornea may be extremely painful. It has several functions that are essential for clear vision: The integrity and functionality of the epithelium [5] and endothelium [6], corneal shape [1], and transparency [1]. The corneal endothelium maintains corneal transparency by regulating water content of corneal stroma. The cornea provides approximately two-thirds of the total refractive power of the eye (Figure 2) [6]; thus, even a small change in corneal contour may result in refractive errors. 

According to the World Health Organization’s global estimation of blindness and visual impairment in 2010, 285 million people were reported to be visually impaired [7]. Corneal diseases are the fourth leading cause of blindness worldwide [7]. Causes of corneal endothelial disease (CED) include endothelial dystrophies, iridocorneal endothelial syndrome, and endothelial dysfunction following cataract surgery and corneal transplantation. Corneal endothelial disease usually presents with a gradual onset of decreased vision. Advanced CED can cause recurrent corneal epithelial erosions, resulting in episodes of severe pain. The corneal endothelium is derived from embryonic neural crest cells [8]. Human corneal endothelial cells (HCECs) have limited proliferative capacity *in vivo* and are suggested to be arrested in the G1-phase of cell cycle [9]. In addition, age-related decrease in corneal endothelial cell density is reported. The mean corneal endothelial cell density decreases from 3600 cells per square millimeter (cells/mm^2^) at age 5 years to 2700 cells/mm^2^ at age 15 years [10]. Further reduction of the central corneal endothelial density in adults is reported at the rate of 0.6% yearly with gradual change in cell shape and size [11]. Corneal endothelial cell density below critical level of approximately 500 cells/mm^2^ results in corneal edema and thereby decreased visual acuity. Significant HCEC loss and inadequate replacement of corneal endothelial cells *in vivo* suggest there is a lack of or inefficient cell division. In corneal endothelial wound healing in humans, the endothelial cells adjacent to the wound enlarge as they elongate and slide to the wound area [12]. At present, transplantation is the only available treatment for diseases affecting the corneal endothelium. There are two main types of corneal transplantation for CED: Penetrating keratoplasty and endothelial keratoplasty. Penetrating keratoplasty refers to the replacement of all corneal layers of the recipient’s cornea with a donor cornea. Selective replacement of the diseased posterior layer of the cornea is called endothelial keratoplasty [13]. The above surgical advancements are, however, hindered by the worldwide scarcity of available healthy donor corneas.

A considerable research effort has been put into developing alternative methods for treatment of CED. The remaining HCECs may be stimulated to proliferate or enhance their function with topical eye drops [14] or cell suspension injection into the anterior chamber [15,16]. Magnetic field-guided *in vitro* cultivated HCEC delivery is thought to attract the cells towards Descemet’s membrane [15,17,18]. However, the possible complications of injection of cell suspension into the anterior chamber, such as an increase in intracellular pressure due to clogging of the trabecular meshwork, should be further investigated before human trials are initiated. Although growth factors may promote corneal endothelial wound healing [19], it does not induce HCEC proliferation [20]. Thus, there is a clinical interest for engineering corneal endothelium for transplantation purposes. With increasing advances in regenerative medicine, several research groups have investigated on expansion of corneal endothelial cells and transplantation of tissue engineered corneal endothelium in experimental animal models [21,22,23,24,25,26,27,28,29,30,31,32,33,34,35,36]. The present review focuses on emerging substrates for improved culturing of HCECs. To provide a background for the current use of substrates, cell sources for tissue engineering of corneal endothelium are also described.

## 2. Cell Sources for Tissue Engineering of Corneal Endothelium

Various efforts have been made to increase the availability of human HCEC lines. These include immortalization of retroviral transduction by simian virus 40 (SV40) T antigen [37,38], Cdk4R24c/CyclinD1 [39], and/or human papilloma virus 16 E6/E7 [40]. Immortalized cells increase the risk of tumor formation, aneuploidy [41], and structural rearrangements [42]. Recently, the establishment of untransfected HCEC line [43] and immortalization of HCECs with human telomerase reverse transcriptase have been explored [44]. However, the limitation of methods not using transfection is immortalization of only a subpopulation of the primary culture.

Although the HCECs have limited proliferative capacity *in vivo*, these cells have the ability to proliferate under *in vitro* culture conditions [18,25,29,30,31,32,36,45,46,47,48,49,50,51,52,53,54,55,56,57,58,59,60,61,62,63,64,65,66,67,68,69,70,71,72,73,74,75,76,77,78,79,80,81,82,83,84,85,86,87,88,89,90,91,92,93,94,95,96,97,98,99,100,101,102,103,104,105,106,107,108,109,110,111,112,113,114,115,116,117,118,119]. Primary HCECs, human HCEC lines, and stem cells have been utilized for tissue engineering of corneal endothelium. Donor corneoscleral rims, which remain after corneal trephination for corneal transplantation, and human cadaver corneas that are unsuitable for corneal transplantation provide sources for primary HCECs. The age of donors used in tissue engineering of corneal endothelium varies substantially in the literature. Primary HCEC cultures have been established from corneas from 8-week-old human embryos [120] up to age 80 [49,62]. The proliferative response of HCECs tends to decrease in older donors [59,64,69,75,121,122,123]. Interestingly, Gao *et al.*, in 2010, were not able to demonstrate a high proliferative rate in human fetal corneal endothelial cells [124]. Regardless of age, human corneal endothelial cells from peripheral areas of the cornea are reported to exhibit a higher replication competency compared to the central area [60,73,121,122,123,124]. The lower proliferative capacity of HCECs from the central area may be due to senescence-like characteristics of central HCECs, including stress-induced premature senescence [122]. In addition, it remains interesting to investigate if there are potential stem like progenitors of corneal endothelium that may have more proliferative capacity to produce more corneal endothelial cells than their progency with limited proliferative capacity in center areas of the growing colonies *in vitro*. Isolation of sphere forming HCECs has in fact been reported vividly and has been considered as a potential source of progenitor cells [72,80,108,125,126,127]. It is possible that such progenitor cells in the central region of the colonies in culture may acquire altered epigenetic modifications which could in turn inhibit their further proliferation or result in their terminal differentiation followed by senescence similar to that was reported with many instances of embryonic stem cell colonies in cell cultures [128].

Stem cells are a potential source for engineering of many organs including corneal endothelium. Organ specific adult stem cells, as well as directed differentiation competent embryonic stem cells, and induced pluripotent stem cells (iPS cells) form such sources. Adult stem cells are suggested to reside in the junction between the peripheral corneal endothelium and anterior part of the trabecular meshwork [129]. Embryonic stem cells [130] have the major advantages due to their characteristics of pluripotency and an unlimited proliferation capacity. However, ethical concerns, immune rejection, and risk of teratoma formation have limited the application of embryonic stem cells in clinical trials. The use of iPS cells in clinical trials is also limited because of bio-safety concerns, epigenetic memory from somatic cells, unintended genomic alterations, and related oncogenesis exacerbated by the use of retroviral or lentiviral transducing vectors. The above said sphere forming HCECs [72,80,108,125,126,127] and also human corneal stromal precursors may represent a potential source for corneal endothelial cells [131]. Other sources of human corneal endothelial-like cells for tissue engineering of corneal endothelium include umbilical cord blood mesenchymal stem cells [132], adipose-derived stem cells [133], and bone marrow-derived endothelial progenitor cells [134]. Functional corneal endothelium tissue engineered from corneal stromal derived stem cells of neural crest origin in humans and mice [131], and corneal endothelial like cells from neural crest origin in rats [135], are also reported. However, there are no specific bio-markers for identification of corneal endothelial cells. Although sodium-potassium adenosine triphosphatase (Na^+^K^+^ATPase) and zonula occludens-1 (ZO-1) are located on the corneal endothelial cell membrane, both are also present in other type of cells. Therefore, the isolation of HCECs from donor corneas has been widely followed.

In 1965, Mannagh *et al.* reported successful expansion of HCECs [45]. Following this report, different isolation techniques and culture media have been introduced to harvest and expand HCECs. At present time, isolation of HCECs technique consists of two steps. At first the Descemet’s membrane is peeled with HCECs, thereafter the peeled membranes undergo enzymatic treatment to dissociate the HCECs. Human corneal endothelial cells have largely been a challenging task to culture and expand. So far, there is no superior culture medium for consistent expanding of HCECs.

## 3. Substrates for Cultivation of Human Corneal Endothelial Cells

*In vitro* expansion of HCECs is challenging, and the cells require native-like favorable growth conditions. The cultivated corneal endothelium is fragile and difficult to handle. Therefore, the use of substrates provides mechanical support during transplantation of *ex vivo* engineered human corneal endothelial sheets. In addition, they may create a favorable microenvironment needed for cellular activity. Ideally, the substrate should mimic Descemet’s membrane in its biological, mechanical, chemical, and physiological characteristics. A spectrum of substrates is used in *in vitro* expansion of HCECs and in reconstruction of human corneal endothelial layer. These include biological, synthetic, and biosynthetic materials (Table 1).

For bioengineering of corneal endothelium the substrate materials should preferably fulfil the following criteria: (i) provide favorable microenvironment for corneal endothelial cellular activity; (ii) provide mechanical support; (iii) promote cell layer-carrier interactions, cell adhesion, and extracellular matrix deposition; (iv) be non-toxic; (v) allow transport of gases, nutrients, and molecules; (vi) be easy to handle during cell layer transport or surgery (endothelial keratoplasty); (vii) be transparent; (viii) be easily reproducible (*i.e.*, (v)–(vii) are applicable for transplantation for tissue engineered corneal endothelial grafts).

The substrate should preferably create desired microenvironment for HCEC viability, cell proliferation, and signaling pathways. The corneal endothelium displays high pump capacity and barrier function *in vivo* in order to maintain the cornea in its relatively dehydrated physiological state. The substrate materials must enable support of these principle functions of HCECs and corneal endothelium. Following transplantation of tissue engineered corneal endothelial graft; the substrate should allow sufficient transport of gases, nutrients and molecules between corneal endothelium and stroma.

The Descemet’s membrane is a specialized basement membrane. After birth, the corneal endothelium secretes Descemet’s membrane consisting of non-banded collagen in physiological conditions [136]. In tissue engineering, it is difficult to reconstruct a substrate that totally mimics complex composition, dynamic nature and multiple function of a native basement membrane. Therefore, it might be beneficial if the substrates are able to stimulate collagen secretion.

The substrates should be easy to reproduce, and either degradable or non-degradable substrates may be used in transplantation. If biodegradable, the substrate dissolution rate must be at a preset value that does not give adverse effect on rest of the eye. As microsurgery and minimal incision operations are increasingly used, the tissue engineered corneal endothelium on substrate should be easy to handle and fold/unfold under the surgery.

There are various substrates applied for cultivated HCECs in experimental models (Table 1). In this review, the substrates are classified for convenience into biological, synthetic, and biosynthetic groups of substrates.

**Table 1:** Cultivation of primary human corneal endothelial cells on different types of substrate.

Main Groups of Substrate	Specific Substrates	Author(s)/Year	Cell Suspension/Sheet on Substrate	Cell Density */Suspension at Time of Seeding on Substrate	Final Cell Density on the Substrate *	Morphology	Phenotype
*Biological Substrates*							
Amniotic membrane	Denuded human AM	Ishino *et al.*, 2004 [68]	Cell suspension (trypsinized)	3285 ± 62	2410 ± 31	Polygonal, uniformly sized cells with cell-cell and cell-AM contact	ZO-1
Decellularized/devitalized corneal materials	Culture flasks + ** human cornea denuded of endothelium	Insler and Lopez, 1986 [29]	Cell suspension (trypsinized)	100 µL of 7.5 × 10^5^ cells	560–1650	–	–
	Culture flasks + human cornea denuded of endothelium	Insler and Lopez, 1991 [30]	Cell suspension (trypsinized)	–	–	–	–
	Culture flasks + human cornea denuded of endothelium	Insler and Lopez, 1991 [31]	Cell suspension (trypsinized)	2000–2200	1000–1600	–	–
	Culture plates + human cornea denuded of endothelium	Chen *et al.*, 2001 [62]	Cell suspension (trypsinized)	1503–2159	1895 ± 178	Polygonal with cell-cell adhesion complexes and gap junction	ZO-1
	Bovine ECM coated culture dishes + human cornea denuded of endothelium	Amano, 2003 [67]	Cell suspension (trypsinized)	Cell suspension 2 × 10^5^ in 2 mL	2380 ± 264	Uniform in size and shape	–
	Bovine ECM coated culture dishes + rat cornea denuded of endothelium and coated with fibronectin	Mimura *et al.*, 2004 [27]	Cell suspension (trypsinized)	300 µL of 1 × 10^6^ cells	2744 ± 337	Polygonal	–
	Bovine ECM coated culture dishes + human cornea denuded of endothelium	Amano *et al.*, 2005 [71]	Cell suspension (trypsinized)	2 mL of 2 × 10^5^ cells	2380 ± 264	*In vivo* morphology with cell-cell contact	–
	Decellularized human corneal stroma	Choi *et al.*, 2010 [90]	Cell suspension (trypsinized)	130–3000	–	Compact cells	ZO-1, Na^+^K^+^ATPase, connexin 43
	Culture plates + decellularized posterior lamellae of bovine cornea	Bayyoud *et al.*, 2012 [137]	Cell suspension (trypsinized)	5 × 10^4^ cells/well	2380 ± 179	Polygonal	ZO-1, Na^+^K^+^ATPase, Na^+^HCO_3_^−^, connexin 43
	Culture plates + decellularized porcine cornea	Yoeruek *et al.*, 2012 [138]	Cell suspension (trypsinized)	–	–	–	–
Lens capsule	Deepithelialized human anterior lens capsule	Yoeruek *et al.*, 2009 [88]	Cell suspension (trypsinized)	5 × 10^4^ cells/well	3012 ± 109	Polygonal	ZO-1, Na^+^K^+^ATPase, connexin 43
	Culture plates + deepithelialized human anterior lens capsule	Kopsachilis *et al.*, 2012 [99]	Cell suspension (trypsinized)	5 × 10^4^ cells/well	2455 ± 284	Hexagonal	ZO-1, Na^+^K^+^ATPase
Natural polymers	Collagen-coated, dextran-based microcarrier beads	Insler and Lopez, 1990 [56]	Cell suspension (isolated cells)	–	–	Cobbelstone	–
	Collagen membranes	Kopsachilis *et al.*, 2012 [99]	Cell suspension (trypsinized)	5 × 10^4^ cells/well	2072 ± 325	Hexagonal	–
	Atelocollagen coated culture dishes + collagen vitrigel	Yoshida *et al.*, 2014 [139]	Cell suspension (trypsinized)	1.3 × 10^6^ cells/well	2650 ± 100	–	–
	Type I collagen sponges	Orwin and Hubel, 2000 [65]	Cell suspension (trypsinized)	–	–	Cobbelstone	–
	Bovine ECM coated culture dishes + type I collagen sheet	Mimura *et al.*, 2004 [28]	Cell suspension (trypsinized)	300 µL of 1 × 10^6^ cells	–	Also fibroblast like cells	–
	Type I collagen coated culture dishes	Choi *et al.*, 2013 [97]	Cell suspension (trypsinized)	–	–	–	ZO-1, Na^+^K^+^ATPase
	Type I collagen coated culture plates	Numata *et al.*, 2014 [110]	Cell suspension (trypsinized)	–	–	Hexagonal	ZO-1, Na^+^K^+^ATPase
	Type IV collagen coated culture dishes	Choi *et al.*, 2010 [90]	Cell suspension (trypsinized)	–	–	Compact	–
	Type IV collagen coated culture dishes	Yamaguchi *et al.*, 2011 [93]	Cell suspension (trypsinized)	6000	–	–	–
	Type IV collagen coated culture dishes	Choi *et al.*, 2013 [97]	Cell suspension (trypsinized)	–	–	–	ZO-1, Na^+^K^+^ATPase
	Type IV collagen coated culture plates	Numata *et al.*, 2014 [110]	Cell suspension (trypsinized)	–	–	Hexagonal	ZO-1, Na^+^K^+^ATPase
	Bovine ECM coated culture plates	Blake *et al.*, 1997 [59]	Cell suspension (trypsinized)	–	–	Hexagonal	–
	Bovine ECM coated culture plates	Yamaguchi *et al.*, 2011 [93]	Cell suspension (trypsinized)	6000	–	–	–
	Fibronectin coated culture plates	Blake *et al.*, 1997 [59]	Cell suspension (trypsinized)	–	–	Hexagonal	–
	Fibronectin coated culture plates	Choi *et al.*, 2010 [90]	Cell suspension (trypsinized)	–	–	Compact	–
	Fibronectin coated culture dishes	Yamaguchi *et al.*, 2011 [93]	Cell suspension (trypsinized)	6000	–	–	–
	Fibronectin coated culture plates	Choi *et al.*, 2013 [97]	Cell suspension (trypsinized)	–	–	–	ZO-1, Na^+^K^+^ATPase
	Fibronectin coated culture plates	Numata *et al.*, 2014 [110]	Cell suspension (trypsinized)	–	–	Hexagonal	ZO-1, Na^+^K^+^ATPase
	FNC coating mix coated culture plates	Choi *et al.*, 2013 [97]	Cell suspension (trypsinized)	–	–	–	ZO-1, Na^+^K^+^ATPase
	Gelatin coated culture flasks	Nayak and Binder, 1984 [50]	Cell suspension (trypsinized)	–	–	Flattened and polygonal	–
	A mixture of laminin and chondroitin sulfate coated culture plates	Engelmann *et al.*, 1988 [51]	Cell suspension (trypsinized)	–	–	Mosaic pattern	–
	Thermoresponsive PIPAAm–grafted surfaces + gelatin discs	Hsiue *et al.*, 2006 [76]	Cell sheet	–	–	Polygonal	ZO-1
	Thermoresponsive PIPAAm–grafted surfaces + gelatin discs	Lai *et al.*, 2007 [32]	Cell sheet	4 × 10^4^ cells	2587 ± 272	Polygonal with cell-cell contact	ZO-1, Na^+^K^+^ATPase
	Type IV collagen coated culture dishes + gelatin hydrogel sheets	Watanabe *et al.*, 2011 [94]	Cell suspension (trypsinized)	3–5 × 10^3^	–	Mosaic pattern with ruffled borders	ZO-1, Na^+^K^+^ATPase, *N*-cadherin
	Laminin-5 coated culture dishes	Yamaguchi *et al.*, 2011 [93]	Cell suspension (trypsinized)	6000	–	–	–
	Laminin coated culture plates	Choi *et al.*, 2013 [97]	Cell suspension (trypsinized)	–	–	–	ZO-1, Na^+^K^+^ATPase
*Synthetic Substrates*							
	Rose chamber	Mannagh and Irving, 1965 [45]	Cell suspension (isolated cells)	–	–	Elongated with cell-cell contact	–
	Tissue culture dishes or flasks	Newsome *et al.*, 1974 [46]	Endothelium-Descemet’s membrane explant	–	–	Flat and polygonal	–
	Culture flasks or Petri culture dishes	Baum *et al.*, 1979 [47]	Endothelium-Descemet’s membrane explant	–	–	Small and uniform in young donors. Large and pleomorphic in older donors	–
	Coverglass of disposable tissue culture chamber	Tripathi and Tripathi, 1982 [49]	Isolation of cells by scraping and Descemet’s membrane explant	–	–	Flattened and hexagonal or polygonal	–
	Culture plates	Blake *et al.*, 1997 [59]	Cell suspension (trypsinized)	–	–	Hexagonal	–
	Collagen type IV coated culture dishes + thermoresponsive PIPAAm–grafted surfaces	Sumide *et al.*, 2006 [74]	Cell sheet	3 × 10^6^ cells/dish	3000	Hexagonal with cilia and microvilli	–
	Thermoresponsive PIPAAm-grafted culture dishes	Ide *et al.*, 2006 [140]	Cell sheet	–	–	Polygonal with cilia and microvilli	–
	Thermoresponsive PIPAAm-grafted culture dishes	Lai *et al.*, 2006 [141]	Cell sheet	4 × 10^4^ cells	2500	Hexagonal	ZO-1, Na^+^K^+^ATPase
	Bovine ECM coated culture dishes + culture plates and culture inserts	Hitani *et al.*, 2008 [25]	Cell sheet	600 µL of 4 × 10^6^ cells	2425 ± 83	Uniformly sized cells	ZO-1, Na^+^K^+^ATPase
	Culture plates	Choi *et al.*, 2010 [90]	Cell suspension (trypsinized)	–	–	Compact	–
	Culture plates	Yamaguchi *et al.*, 2011 [93]	Cell suspension (trypsinized)	6000	–	–	–
	Culture plates	Kopsachilis *et al.*, 2012 [99]	Cell suspension (trypsinized)	5 × 10^4^ cells/well	2507 ± 303	Hexagonal	–
	Culture plates	Choi *et al.*, 2013 [97]	Cell suspension (trypsinized)	–	–	–	ZO-1, Na^+^K^+^ATPase
*Biosynthetic Substrate*							
****	FNC coating mix coated culture dishes + FNC coated RAFT + collagen gel (compressed plastic and type I collagen)	Levis et al., 2012 [101]	Cell suspension (trypsinized)	2000	1941	Polygonal	ZO-1, Na^+^K^+^ATPase

Notes: AM: Amniotic membrane; DM: Descemet’s membrane; ECM: Extracellular matrix; FNC Coating Mix^®^: A commercial available coating mixture consisting of fibronectin, collagen and albumin; Na^+^HCO_3_^−^: Sodium bicarbonate; Na^+^K^+^ATPase: Sodium-potassium adenosine triphosphatase; PIPAAm: Poly (*N*-isopropylacrylamide); Pos: Positive; RAFT: Real Architecture For 3D Tissues; ZO-1: Zona occludens. * cell density in cells/mm^2^ if otherwise not stated; ** change of substrate for cultivation of cells.

### 3.1. Biological Substrates

#### 3.1.1. Amniotic Membrane

Amniotic membrane (AM) is a membrane composed of collagen type IV similar to basement membrane of conjunctiva but not cornea [142]. The anti-inflammatory [143] and non-immunogenic [144] properties of AM are believed to be important factors that make it a suitable substrate. The AM is used in treatment of different ocular surface diseases, and it is applied as substrate for limbal transplantation in patients with limbal stem cell deficiency [5]. Ishino *et al.* used denuded AM as a substrate for cultivated HCECs and transplanted onto rabbit corneas denuded of corneal endothelium and Descemet’s membrane [68]. The authors demonstrated that the corneal endothelial cell density and function of reconstructed corneal endothelial graft were similar to normal corneas. However, the tissue-engineered grafts consisting of HCECs sheet on AM had some edema. In another study, the basement membrane of AM was used as a carrier for transplantation of cultivated cat corneal endothelial cells on cat cornea denuded of Descemet’s membrane and endothelium [145]. The cultivated cells predominantly displayed hexagonal shape, and the reconstructed corneal endothelial layer maintained corneal graft thickness and remained transparent for six weeks.

Although AM provides good biocompatibility, dependency on donor tissue is a limitation. However, AM displays several challenges for clinical use, and thus efforts to identify alternative culture substrates should be encouraged. First, it is semi-opaque; second, preparation is time-consuming; third, there is possible transfer of pathogens from AM; and fourth, inter-donor and intra-donor variations and rate of biodegradability may influence the outcome of its clinical use [146].

#### 3.1.2. Decellularized/Devitalized Corneal Materials

The feasibility of using devitalized corneas or corneas denuded of endothelium as substrate for HCECs is studied extensively [27,29,30,31,62,67,71,97,137,138]. They are applicable without substantial redesign as they provide the desired shape, mechanical support, and transparency. Reconstructed human corneal endothelial graft with *in vitro* cultivated HCECs seeded on decellularized human corneal stroma expressed ZO-1, Na^+^K^+^ATPase and connexin 43. Proulx *et al.* studied the function of tissue engineered corneal endothelium [33]. In experimental animal models they transplanted tissue engineered corneal endothelial grafts consisting of cultivated feline corneal endothelial cells on devitalized human cornea denuded of endothelial cells. The follow-up time after transplantation was only 7 days. In this study, 9 of 11 reconstructed corneal endothelial grafts were clear at the end of the follow-up time. The pump function of the reconstructed corneal endothelial graft must have remained functional in order to maintain the cornea transparent. In addition, the reconstructed corneal endothelial layers expressed proteins related to function such as ZO-1 and Na^+^K^+^ATPase and sodium bicarbonate (Na^+^HCO_3_^−^) transporter [33]. The same research group performed ultrastructural and immunohistochemical studies of cultivated feline corneal endothelial layer on devitalized cornea [147]. Scanning and transmission electron microscopy demonstrated a monolayer of corneal endothelium, and the tissue engineered endothelium expressed function related proteins including ZO-1 and Na^+^K^+^ATPase and Na^+^HCO_3_^−^ transporter.

Bayyoud *et al.* seeded *in vitro* expanded HCECs on devitalized posterior corneal stromal lamellae. The reconstructed corneal endothelial graft had intact barrier and expressed positive staining for sodium-potassium pump (Na^+^K^+^ATPase), membrane transporter (Na^+^HCO_3_^−^), tight junction (ZO-1), gap junction (connexin 43), and extracellular matrix protein (collagen VIII) [137].

Current methods to decellularize or devitalize cornea include scraping off corneal endothelium mechanically [67,71], use of chemicals [62,137], or freeze/thaw method [33,147]. High-hydrostatic pressurization is an alternative technique to decellularize cornea [148]. However, the following are some inherent technicalities to be aware of using this approach. First, resident viable keratocytes may potentially give raise to fibroblastic contamination. Second, biological tissues may transfer infections. Third, stroma from donor corneas does not reduce the dependency of donor tissues.

#### 3.1.3. Lens Capsule

The human anterior lens capsule has been evaluated as potential substrate for tissue engineered corneal endothelium. Yoeruek *et al.* received human anterior lens capsule from patients who had undergone cataract surgery [88]. They seeded HCECs on de-epithelialized anterior lens capsule and demonstrated that the HCECs grew to confluency. The *in vitro* bioengineered corneal endothelium strongly expressed staining for ZO-1, Na^+^K^+^ATPase, and connexin 43. Kopsachilis *et al.* compared three different substrates; these included de-epithelialized human anterior lens capsule, collagen membrane, and polystyrene culture plates [99]. They obtained human anterior lens capsule of a mean diameter of 10 mm from cornea donors. The cultivated cells displayed hexagonal morphology in all groups, and the cells formed a monolayer of corneal endothelium at two weeks. They reported higher cell density on anterior lens capsule and culture plates in comparison to collagen membrane (Table 1). However, no statistically significant difference in cell density was shown among all three groups. Although the de-epithelialized human anterior lens capsule is a biocompatible substrate, it does not reduce donor dependency. The diameter of anterior lens capsule following capsulorhexis in cataract surgery is approximately half the size needed for a carrier for cultivated corneal endothelium for endothelial keratoplasty.

#### 3.1.4. Natural Polymers

Extracellular protein coatings are composed of single proteins (e.g., collagen, gelatin) or combination of different proteins (e.g., FNC coating mix^®^). Although the exact components and composition of the coatings are known, the biological activity of HCECs on these coatings varies. The coating proteins influence HCEC adhesion, proliferation, morphology, and function of HCECs. There are many different types of coating materials available for expansion of HCECs. These include collagen [28,56,65,74,90,93,97,99,110,139], fibronectin [59,90,93,97,110], gelatin [32,50,76,94], laminin [93,97], extracellular matrix (ECM) from cultured bovine corneal endothelial cells [25,27,59,67,71,93], a mixture of laminin and chondroitin sulfate [51], and a mixture of fibronectin, collagen, and albumin (FNC Coating Mix^®^) [97,101].

Choi *et al.* evaluated adhesion, proliferation, and phenotypic maintenance of HCECs on ECM coated culture plates [97]. They studied collagen type I, collagen type IV, fibronectin, laminin, and FNC coating mix. The HCECs expressed a number of integrin genes (integrin α1, α2, α3, α*v*, β1 and β5), but not integrin gene β3. High expression of integrin genes supports HCEC binding to ECM. Although cells on collagen type IV and fibronectin showed the highest expression and cells on collagen type I exhibited the least expression, there were no statistically significant differences. Compared to uncoated control plates, HCECs adhered more tightly to culture plates coated with coating proteins such as collagen I, collagen IV, fibronectin, and FNC coating mix. The authors also investigated the cell adhesion strength, and showed that all the coating proteins increased the adhesion strength compared to uncoated controls, except for laminin. They were able to demonstrate that HCECs could grow into a confluent layer in a week on all ECM tested, including uncoated culture plates. Gene expression of ZO-1 and Na^+^K^+^ATPase was found in all conditions, but Na^+^K^+^ATPase expression was significantly higher in collagen type I, fibronectin and laminin coated culture plates [97]. In a previous study of Choi *et al.*, it was demonstrated that proliferation of HCECs on fibronectin coated culture plates was significantly higher on day 2 after seeding compared to collagen type IV coated culture plates and uncoated culture plates [90]. On day 4 after seeding, however, there was no significant difference in the growth rate in any of the experimental groups.

Yamaguchi *et al.* studied HCEC adhesion and proliferation in the presence of recombinant laminin-5 [93]. Their results showed significantly higher adhesion of HCECs on recombinant laminin-5 coated dishes compared to uncoated control culture dishes. Furthermore, HCECs did not proliferate on collagen type IV coated culture dishes, and the number of adherent HCECs on laminin-5 coated culture dishes increased 1.5 times after 7 days of cell culture.

In few studies gelatin as substrate for HCECs was evaluated [50,76,94]. Hsiue *et al.* were able to demonstrate that gelatin discs dissolved and the HCEC sheet was adherent to posterior part of corneal stroma two weeks after transplantation of HCEC sheet [76]. Silkworm fibroin can be prepared as a transparent membrane and used as carrier for cultivated corneal endothelial cells [149]. However, higher cell density of B4G12 cell line was achieved on uncoated tissue culture compared to on fibroin. Human corneal endothelial cells grew to confluency with polygonal morphology only on collagen type IV coated fibroin [149].

Extracellular matrices from cultured bovine corneal endothelial cells are used as coating material for *in vitro* cultivation HCECs [25,27,59,67,71,93]. In a study the HCECs were cultured initially on bovine ECM coated culture dishes following seeding of the cells on type I collagen sheet [28]. The HCEC sheet was reported to also have cells with fibroblastic-like morphology. Extracellular matrices produced by bovine corneal endothelial cells may be reservoir for progelatinase A, a matrix metalloproteinase, which is important for turnover of ECM and is involved in inflammation, wound healing, angiogenesis, and metastasis [150].

Studies were carried out by using collagen type I and IV as a substrate for HCECs [74,90,93,97,110]. Cultivated monkey corneal endothelial cells were further cultured on collagen type I carrier for 4 weeks and transplanted into monkeys. The cultivated corneal endothelial layer produced confluent monolayer expressing ZO-1 and Na^+^K^+^ATPase. The transplanted tissue-engineered corneal endothelial graft remained clear and had an endothelial cell density of 1992 to 2475 cells/mm^2^ on examination using *in vivo* specular microscopy six months after transplantation [35].

Cultured HCECs on collagen sheets composed of cross-linked collagen type I were transplanted into rabbits. Pump function was evaluated using Ussing chamber and ouabain, a Na^+^K^+^ATPase inhibitor. The results showed that the cultured HCECs on collagen sheets maintained 76%–95% of pump function of human donor corneas [28].

The difference in adhesion, proliferation, and phenotype displayed by HCECs on the same type of coating in different studies can be related to different culture techniques and media used. However, further studies should be conducted to assess the consistency of the different types of coatings. The use of these coatings in clinical setting remains to be rigorously verified as the coatings are derived from animals.

### 3.2. Synthetic Substrates

Synthetic polymers have the advantage of high purity with known chemical composition, structure and properties. They can be reproduced at controlled conditions with known mechanical and physical properties. Coated hydrogel lens was used as carrier for cultivated kitten and rabbit corneal endothelial cells, and these constructs were transplanted into adult cats and rabbits with induced corneal edema, respectively. The transplanted corneas became clear within three days after transplantation in both cats and rabbits, and the cornea remained clear for 50 days in cats and 40 days in rabbits [23].

In few studies the HCECs were cultured on plastic culture plates without coating [45,46,47,49]. These studies do not reveal details of adhesion and proliferation profiles of HCECs. The cyclic dimers of glycolic and lactic acids are monomers used in production of biomedical devices. Glycolic and lactic acids are by-products of metabolic pathway in normal physiological conditions. Therefore, they are regarded as highly biocompatible with minimal systemic toxicity. Poly(lactic acid) (PLLA) and poly(lactic-*co*-glycolic acid) (PLGA) are synthetic polymers extensively studied owing to their biocompatibility and biodegradability [151]. Hadlock *et al.* seeded *in vitro* expanded rabbit corneal endothelial cells on PLLA and PLGA [152]. In tissue culture conditions the cells grew into confluency on the synthetic materials and stained for ZO-1 along the lateral cell borders.

Synthetic polymers are used commonly as drug delivery devices. In few ocular diseases dexamethasone can be delivered into the vitreous cavity as an implant. Ozurdex, consisting of dexamethasone and PLGA with hydroxypropyl methylcellulose, is injected intravitreally in patients with e.g., macular edema secondary to retinal vein occlusion. Poly(lactic-*co*-glycolic acid) polymer matrix degrades slowly to lactic and glycolic acids meaning the final degradation products are water and carbon dioxide [153]. Another dexamethasone delivery device, Surodex, consisting of PLGA with hydroxypropyl methylcellulose is inserted into the anterior chamber following cataract surgery to treat postoperative inflammation. In a comparative single-masked parallel-group study, Wadood and coauthors compared the safety and efficacy of dexamethasone eye drops and Surodex inserted into the anterior chamber in patients following phacoemulsification cataract extraction and posterior chamber intraocular lens implantation [154]. Out of 19 patients in this study, 11 patients received Surodex. Surodex remnants were present in all eyes at 60-day post-operative control, and in 3 patients the traces of remnants were present at 32–36 months. However, no significant complications were reported during the follow-up time of 3 years. The authors reported peripheral anterior synechias of less than 1 clock hour at the site of Surodex implantat in 1 patient, and they regarded this as an adverse event. One patient developed high intraocular pressure after Surodex implantation. The authors considered the patient to be a steroid responder. The intraocular pressure normalized without treatment during the follow-up time of 36 months. Although PLGA is considered to be well-tolerated by patients when inserted into the anterior chamber or vitreous cavity, the removal of device in e.g., cases of endophthalmitis remains a major concern due to residing remnants.

In the early phase of cultivation of HCECs, adherence of the cells to the substrate is of great importance to initiate cell growth, while detachment of an intact and confluent cell layer in a later phase is necessary for transplantation purposes. Stimuli-responsive polymers have the ability to change their molecular structures or physicochemical properties according to the variation in the environment they are in. The design of these polymers with associated processes is highly specialized. Major changes, such as alteration in the shape, transparency and permeability to water, can be achieved by a small stimulus, such as change in temperature, pH or wavelength of light.

Research groups have cultivated HCECs on culture dishes grafted with temperature-responsive polymer poly(*N*-isopropylacrylamide) (PIPAAm) which reversibly alter its hydrophobicity/hydrophilicity dependent on incubation temperature [32,74,76,140,141]. They have the advantage of providing both initial cell adhesion and later cell layer detachment. At 37 °C the seeded cells adhere and proliferate on hydrophobic PIPAAm-grafted surfaces. The HCEC sheet detaches from PIPAAm-grafted culture dishes as surfaces become hydrophilic when temperature is reduced below the lower critical solution temperature of 32 °C. A circular portion of 18 mm in diameter in the center of 35 mm of culture dishes was grafted with temperature responsive polymer, PIPAAm [74,76]. The *in vitro* cultivated HCECs were seeded on PIPAAm-grafted culture dishes, and the cells reached confluency in 1–3 weeks [32,74,76,140,141]. The gross appearance of confluent HCEC layer on hydrophobic PIPAAm-grafted surfaces was whitish gray, and the authors related this to accumulation of ECM [32]. Upon reduction of incubation temperature from 37 to 20 °C, the HCEC sheets detached from culture dish surfaces within 45–60 min [32,74,140,141]. Although the HCEC sheets detached as single contiguous layers, their surfaces were reported as wrinkled by Lai *et al.* [140] and as having a white paper-like texture by Hsiue *et al.* [76]. The monolayered cell sheets expressed ZO-1 [32,76,140,141] and Na^+^K^+^ATPase [32,74,141] proteins. Deposit of ECM on basal surface of HCEC sheets were observed [32,74,76,140,141], and the ECM components, collagen type IV and fibronectin, were detected by immunostaining [140]. Scanning electron microscopy micrographs showed polygonal cells with cellular interconnections [74,76,140,141] and microvilli and cilia [74,140], and transmission electron microscopy micrographs revealed abundant cytoplasmic organelles, rough endoplasmic reticulum and mitochondria [32,140]. However, Hsiue *et al.* demonstrated the absence of clear cell boundaries [76].

In two studies, the harvested HCEC sheets from PIPAAm-grafted surfaces were immediately transferred to gelatin disc carriers (7 mm in diameter and 700–800 µm in thickness) [32,76]. The reconstructed corneal endothelium was transplanted into experimental rabbit models denuded of corneal endothelium. The gelatin discs dissolved in two weeks, and the corneas transplanted with reconstructed corneal endothelium were clear with near normal corneal thickness at four weeks [76]. In rabbits transplanted with tissue engineered corneal endothelium, the corneal thickness increased to 892 µm at post-operative day 1, and then decreased to near normal corneal thickness of approximately 500 µm at post-operative day 168 [32]. Sumide *et al.* transplanted HCEC sheet attached to cornea denuded of corneal endothelium and Descemet’s membrane into rabbit models [74]. Control rabbits underwent all procedures except for having HCEC sheet on corneal button. Minimal corneal edema was reported in rabbits in HCEC sheet transplant group at day 7. In contrast, the corneas were opaque in control group. The average corneal thickness in HCEC sheet transplant group was significantly lower compared to control group at day 7. Even though the stimuli-responsive polymers are investigated extensively, their role in corneal endothelial layer transplantations and the effect of the temperature change on the HCEC bioactivity remains to be investigated.

### 3.3. Biosynthetic Substrates

Substrates made from a mixture of natural and synthetic polymers are referred to as biosynthetic substrates in this review. Gao *et al.* evaluated biocompatibility and biodegradability of substrate composed of hydroxypropyl chitosan, gelatin, and chondroitin sulfate [155]. Scanning electron microscopy images revealed a porous structure without fibrils, and the light transmission (wavelength ranging 400–800 nm) measurements through the substrate showed transmittance of more than 90%; both indicating the membrane transparency. They demonstrated comparable or better glucose permeability through the substrate in comparison to native corneas. Cultivated rabbit corneal endothelial cells on this substrate reached confluency on day 4, and displayed characteristic cobblestone appearance. Histocompability and biodegradability were assessed by implanting the substrates into skeletal muscle of rats. Sign of inflammation was seen during post-mortem examination at the interface between the host tissue and substrate even at the end of observation period of 2 months. Degradation of substrate was observed from day 30.

Plastic compressed collagen gels [101] and a blending of chitosan and polycaprolactone [156] may give the necessary mechanical strength as a carrier. Synthetic polymers have the advantage of being reproduced under controlled conditions with known mechanical and physical properties. Different ratio of chitosan and polycaprolactone in a substrate were examined. A composition of 75% chitosan and 25% polycaprolactone supported cultivation of bovine corneal endothelial cells and gave the necessary mechanical strength of a substrate. The cells reached confluency on day 7 and expressed ZO-1 protein on substrate composed of chitosan and polycaprolactone at raio of 75:25 [156].

Plastic compressed collagen type I, termed Real Architecture For 3D Tissues (RAFT), can be easily reproduced and trephined into the size required [101]. Scanning and transmission electron microscopy imaging revealed a confluent monolayer of corneal endothelial cells on RAFT. Human corneal endothelial cells cultivated on RAFT stained for ZO-1 and Na^+^K^+^ATPase proteins [101].

The synthetic polymers degrade slowly, and hence potential adverse effects on the eyes over long time course remains to be investigated. Biosynthetic substrate is reported to give raise to inflammation in experimental animal models [155]. Therefore, histocompability studies should be performed before use of biosynthetic substrates in humans.

## 4. Conclusions and Future Perspective

It is obvious to date that the development and utility of different substrates in tissue engineering of corneal endothelium is slowly evolving. Functional realization of the bioengineered corneal endothelium has not yet been optimal due to the current limited knowledge of molecular mechanism of proliferation of HCECs and their associated inter- and intra-signaling pathways that maintain the corneal endothelial tissue homeostasis. Ideal substrates for cultivation of HCECs should mimic Descemet’s membrane in molecular, physiological and mechanical terms. Therefore, it is essential to have thorough molecular and functional insights into the microenvironment of human corneal endothelium *in vivo* and engineer such characteristics into the deriving HCEC grafts. Identification of specific marker(s) of HCEC will be extremely advantageous in optimizing differentiation of large numbers of HCECs from a variety of available cell sources. In addition, even perhaps the patient specific iPS cells with the eventual goal of prospectively circumventing the need for increasingly limiting donor corneas. Finally, though the preliminary xenotransplantation studies appear promising, focused research on the discovery and derivation of suitable substrates, optimization of HCEC culture techniques and identification of specific marker(s) of HCESs appears very valuable before any bioengineered human corneal endothelial graft is used clinically.
